# Prognostic value of preoperative inflammatory markers in resectable non-small cell lung cancer: a multi-center retrospective study based on logistic regression and machine learning techniques

**DOI:** 10.3389/fmed.2026.1771545

**Published:** 2026-04-22

**Authors:** Xinying Cai, Dongqi Lin, Siqi Wang, Jingwen Li, Xianhui Zhou, Hongtao Liu

**Affiliations:** 1 School of Medicine, Xiamen University, Xiamen, China; 2 Clinical Research Center, Shantou Central Hospital, Shantou, China; 3 Department of Thoracic Surgery, Guangzhou Institute of Cancer Research, The Affiliated Cancer Hospital, Guangzhou Medical University, Guangzhou, China; 4 Department of Cardiothoracic Surgery, The Second Affiliated Hospital of Shantou University Medical College, Shantou, China; 5 The Department of Neurosurgery, The First Hospital of Qiqihar, Qiqihar, China; 6 Xinjiang Key Laboratory of Cardiac Electrophysiology and Remodelling, The First Affiliated Hospital of Xinjiang Medical University, Urumqi, China; 7 Department of Pacing and Electrophysiology, The First Affiliated Hospital of Xinjiang Medical University, Urumqi, China; 8 Department of Pharmacy, The First Hospital of Hebei Medical University, Shijiazhuang, China

**Keywords:** molecular minimal residual disease, non-small cell lung cancer, resectable surgery, retrospectively analyze, systemic inflammatory

## Abstract

**Purpose:**

This study aimed to assess the prognostic significance of the neutrophil-to-lymphocyte ratio (NLR), platelet-to-lymphocyte ratio (PLR), systemic immune-inflammation index (SII), and systemic inflammation response index (SIRI), measured 15 days prior to surgery, in patients undergoing primary resection for NSCLC.

**Methods:**

We retrospectively analyzed data from 460 NSCLC patients treated at two comprehensive hospitals, along with another 50 patients for external validation. Optimal cut-off values for NLR, PLR, SII, and SIRI were determined. Kaplan–Meier analysis was performed to estimate overall survival (OS) and progression-free survival (PFS). Univariate and multivariate Cox regression analyses were conducted to identify independent prognostic factors. To evaluate and validate the reliability of prognostic factor selection, three commonly used machine learning methods—LASSO, Random Forest, and Support Vector Machine (SVM)—were applied. The systemic inflammatory markers were subjected to external validation in an independent cohort, encompassing discriminative analysis, calibration analysis, and clinical decision curve analysis (DCA), with a final evaluation of the SIRI’s underlying rationale.

**Results:**

The optimal cut-off values for NLR, PLR, SII, and SIRI were 3.470, 186, 853.71, and 1.66, respectively. Kaplan–Meier curves demonstrated that elevated levels of all four markers were significantly associated with poorer OS and PFS. The 3- and 5-year OS rates were 83.6 and 72.6%, while the 3- and 5-year PFS rates were 82.4 and 75.0%, respectively. Univariate analysis identified several factors significantly associated with survival, including inflammatory markers, smoking, antibacterial use, lymphatic metastasis, radiotherapy, intraoperative blood loss, and preoperative albumin levels. Multivariate analysis further revealed that lymphatic metastasis, antibacterial use, NLR, and SIRI were independent predictors of OS, whereas lymphatic metastasis, radiotherapy, antibacterial use, NLR, and SIRI were independently associated with PFS. Consistently, machine learning methods also highlighted NLR and SIRI as reliable independent prognostic indicators in patients with resectable NSCLC. SIRI showed good discrimination with a concordance index of 0.803 (95% CI: 0.644–0.962) for external validation.

**Conclusion:**

Preoperative systemic inflammatory markers, particularly SIRI, are strong and independent prognostic indicators of PFS in patients with resectable NSCLC. Among these, a low SIRI may provide superior risk stratification for identifying high-risk patients and informing individualized treatment strategies.

## Graphical abstract

**Figure:**
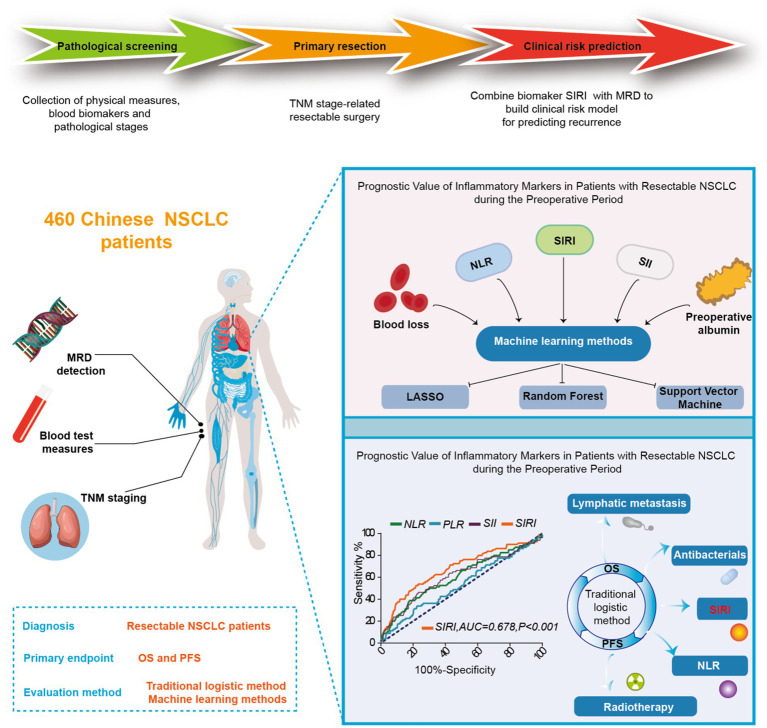

## 1 Introduction

Non-small cell lung cancer (NSCLC) is a highly recurrent malignancy that poses a serious threat to patients’ lives. Globally, approximately 2.2 million new cases of lung cancer are diagnosed each year (1), and 75% of patients die within 5 years of diagnosis (2). Surgery remains the primary curative option for early-stage NSCLC; however, more than 50% of patients experience disease recurrence following surgery alone (3). Among patients with resected NSCLC, pathological staging remains the most accurate prognostic factor and is still considered the gold standard for risk assessment.

Recent advancements in screening technologies, along with the development of systemic therapies, have significantly improved the efficacy of NSCLC treatment following radical resection. In particular, the detection of molecular minimal residual disease (MRD) (4) has enhanced the ability to predict prognosis and guide subsequent treatment strategies, making it a valuable supplementary tool to TNM staging and immunotherapy in the post-resection management of NSCLC. In early-stage NSCLC patients who have undergone radical resection, a positive MRD result is associated with a high risk of recurrence. Current guidelines recommend MRD monitoring every 3 to 6 months (5). However, studies have shown that MRD detection technologies—including tumor-informed assays, customized Next Generation sequencing (NGS) panels, tumor-agnostic approaches, and multi-omics platforms—can impose a significant economic burden on patients. Moreover, MRD research is still in its developmental phase, with substantial variability in MRD definitions and detection methods across studies, leading to challenges in its standardized clinical application. Therefore, there is an urgent need to identify convenient, cost-effective, and reliable prognostic biomarkers that can complement TNM staging, postoperative immunotherapy and MRD detection, thereby enhancing prognostic prediction following primary NSCLC resection.

Previous studies have demonstrated a significant association between postoperative pneumonia and overall survival in patients with lung cancer (6). More recently, we reported that postoperative pulmonary inflammation, assessed 4 months after surgery, was significantly associated with lung cancer recurrence within one year (LRO). Postoperative pneumonia may reflect an elevated immune cell count; however, a high immune cell count does not necessarily indicate the occurrence of pneumonia. Inflammation and immune surveillance are widely recognized as key hallmarks in cancer development. Emerging evidence has confirmed that inflammation plays a critical role in cancer initiation, progression, and metastasis (7). While genetic mutations in cancer cells are regarded as the spark that ignites tumor development, inflammation acts as the fuel that sustains and accelerates this process (8). Moreover, immune cells such as neutrophils, monocytes, and lymphocytes—and their absolute counts—have been identified as prognostic indicators in various tumor models (9). Building on this, several novel systemic inflammatory markers—including the neutrophil-to-lymphocyte ratio (NLR), platelet-to-lymphocyte ratio (PLR), systemic immune-inflammation index (SII), and systemic inflammation response index (SIRI), which integrate different components of the inflammatory response—have shown promise as prognostic predictors across multiple cancer types (10). Although inflammatory markers have been extensively used to predict prognosis in various malignancies (11–13), studies specifically focusing on NLR, PLR, SII, and SIRI in non-small cell lung cancer (NSCLC) remain limited. Existing research has primarily targeted patients with unresectable, locally advanced NSCLC undergoing chemoradiotherapy (10).

In recent years, the use of inflammatory markers in tumor prognosis has gained increasing attention in cancer research. However, investigations into the prognostic significance of preoperative inflammatory markers in patients with resectable NSCLC remain limited. Notably, all the aforementioned inflammatory markers are derived from routine blood tests, which are inexpensive, convenient, and reliable—making them highly promising for widespread clinical application. Therefore, we aimed to evaluate the prognostic value of preoperative NLR, PLR, SII, and SIRI in patients undergoing primary NSCLC resection. It also aims to evaluate the traditional logistic regression and machine learning approaches to compare their statistical power and predictive accuracy.

## 2 Patients and methods

### 2.1 Patients and data collection

**Figure 1:**
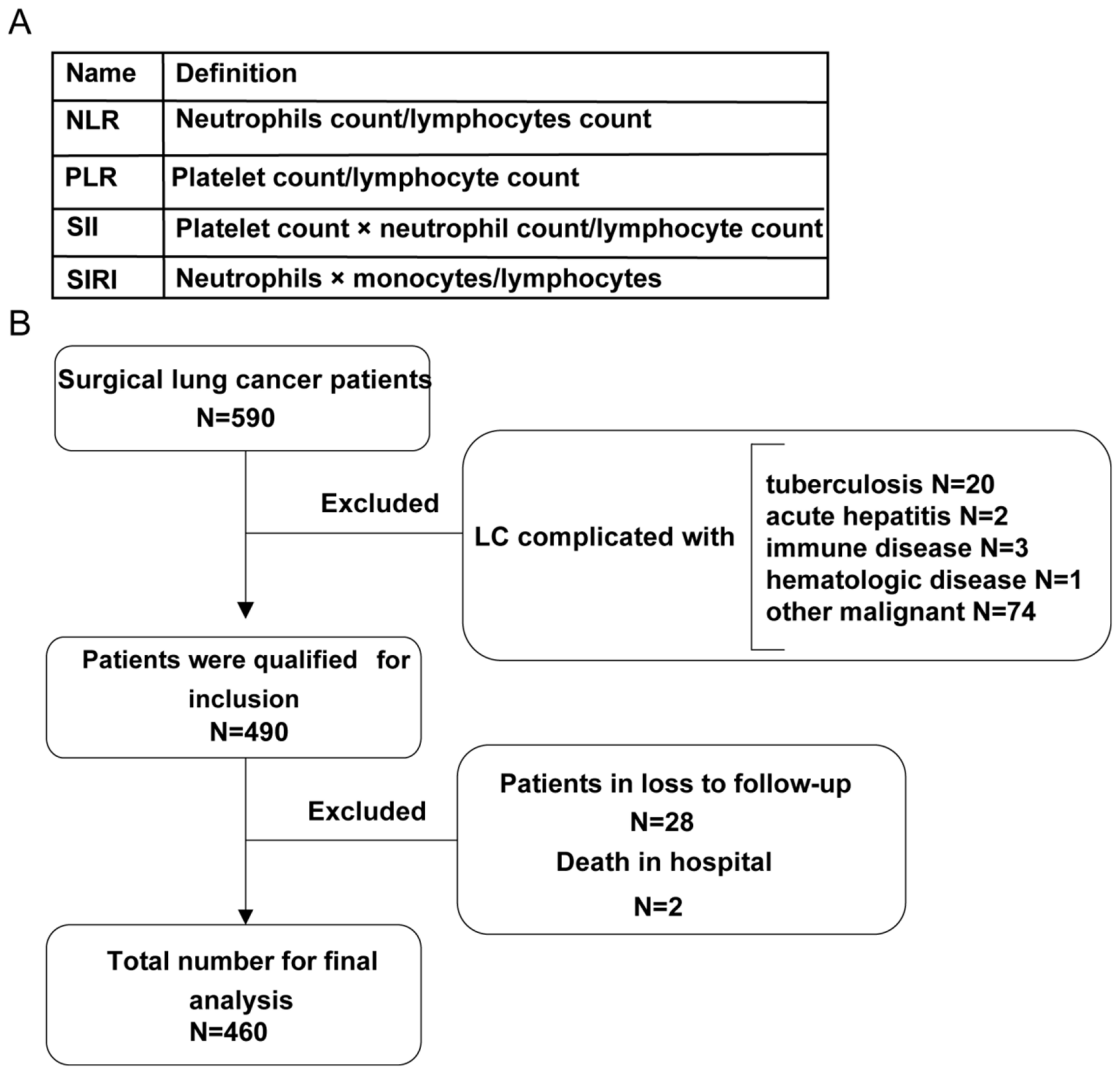
**(A)** The standardized definitions of inflammatory markers. **(B)** The flowchart of data collection based on systemic variables. LC, non-small cell lung cancer.

This study included patients with NSCLC who underwent complete surgical resection at two comprehensive hospitals between 1st/April/2014 and 31st/March/2021. The last follow-up date was 31st/March/2022. An additional cohort of 50 NSCLC patients from Shantou Central Hospital, enrolled between April 1, 2021, and December 31, 2022, was included for external validation. These data were accessed for research purposes in DD/MM/YYYY format. Inclusion criteria were as follows: first histologically confirmed primary NSCLC without distant metastases, staged according to the 9th edition of the American Joint Committee on Cancer (AJCC) staging manual (14); and availability of inflammatory marker values calculated from routine blood test results. At the time of initial diagnosis, all patients were aged 18 years or older and had undergone comprehensive preoperative assessments, including chest computed tomography (CT), abdominal ultrasonography, whole-body bone scintigraphy, and brain magnetic resonance imaging (MRI). Hematological data, including complete blood count and biochemical tests, were obtained within 15 days prior to surgery. Exclusion criteria included: (1) presence of other serious comorbidities such as severe cardiac, hepatic, or renal dysfunction; (2) history of other malignant tumors; (3) acute infectious or hematologic diseases; (4) incomplete medical records or failure to complete prescribed treatment; (5) loss to follow-up. This study was approved by the Ethics Committees of the Second Affiliated Hospital of Shantou University Medical College (Approval No. 2021–38), and was conducted in accordance with the Declaration of Helsinki. A total of 460 eligible patients were ultimately included in the study. Every participant who participated in this study provided informed consent in writing. All of the data were anonymized prior to the data. Our research only recruited adults but not included children. Patient data, including age, sex, smoking history, preoperative comorbidities, and medications, were retrospectively retrieved from the electronic medical record system. Intraoperative data included the type of NSCLC surgery, TNM stage, histological subtype, presence of lymphatic metastasis, operation time, and intraoperative blood loss. Postoperative data encompassed hospital stay duration, intensive care unit (ICU) stay, wound infection, failed extubation, reoperation due to hemorrhage, and postoperative treatments. Laboratory data collected included white blood cell count and serum albumin level. Pretreatment peripheral blood leukocyte (PBL) biomarkers included absolute counts of neutrophils, monocytes, platelets, and lymphocytes. Inflammatory markers were calculated according to the standardized definitions (Figure 1A).

### 2.2 Follow-up

Patients were followed from the date of diagnosis until 31st/ March /2022, or the date of death, whichever occurred first. The primary endpoint of the study was overall survival (OS), defined as the time from diagnosis to death from any cause or the last follow-up. The secondary endpoint was progression-free survival (PFS), defined as the time from diagnosis to disease progression, death, or the last follow-up, whichever occurred first. Postoperative follow-up included contrast-enhanced CT and PET-CT. Recurrence was defined as the appearance of new locoregional or distant lesions on imaging after surgery. All suspected recurrences were confirmed histopathologically: bronchoscopic biopsy was performed for endobronchial or airway-accessible lesions, and CT-guided percutaneous biopsy for peripheral lesions.

### 2.3 Statistical analysis on logistic regression

Statistical analyses were conducted using SPSSAU 2016–2021 (Qing-Si Technology Ltd., Beijing, China). In accordance with previous studies, cut-off values for inflammatory markers were determined using receiver operating characteristic (ROC) curves, which objectively assess the sensitivity and specificity of different thresholds (15, 16). In this study, the optimal cut-off values were selected based on the Youden Index. The chi-square test was used to compare baseline characteristics between low and high groups for NLR, PLR, SII, and SIRI. Kaplan–Meier (K-M) survival curves were generated to compare OS and PFS between groups. Univariate and multivariate Cox proportional hazards models were applied to identify independent prognostic factors, with hazard ratios (HRs) and 95% confidence intervals (CIs) reported. A *p*-value < 0.05 was considered statistically significant. Predictors identified as statistically significant in multivariate Cox regression analysis (*p* < 0.05) or consistently selected by all three machine learning algorithms were incorporated into the final nomogram. Calibration analysis used the Hosmer-Lemeshow test, and a calibration curve was plotted with predicted probabilities on the x-axis and observed probabilities of progression on the y-axis. DCA identified the risk threshold range for clinical utility.

### 2.4 Statistical analysis on machine learning techniques

To identify key variables associated with two dependent outcomes—PFS and OS—we employed three machine learning algorithms: LASSO, Random Forest (RF), and Support Vector Machine (SVM), implemented using the “glmnet,” “randomForest,” and “e1071” packages in R, respectively (17–19). LASSO (Least Absolute Shrinkage and Selection Operator) regression is a robust and widely used method for analyzing high-dimensional data. By applying L1 regularization, LASSO automatically performs variable selection by shrinking less informative coefficients to zero, thereby simplifying the model and reducing the risk of overfitting. Random Forest, an ensemble learning algorithm based on decision trees, enhances predictive accuracy through bootstrap aggregation (bagging). It ranks feature importance using metrics such as the percentage increase in mean squared error (%IncMSE) and the increase in node purity (IncNodePurity), enabling identification of the most predictive variables. Additionally, SVM, a supervised learning algorithm well-suited for classification and regression in high-dimensional spaces, was employed for feature selection. By constructing optimal hyperplanes that maximize the margin between classes, SVM highlights variables with strong discriminative power. In this study, recursive feature elimination (RFE) was used in combination with SVM to further refine the selection of hub variables predictive of PFS and OS.

## 3 Results

### 3.1 Patient characteristics

In this study, we retrospectively analyzed 590 cases from two comprehensive hospitals. Of these, 130 patients did not meet the inclusion criteria and were excluded. The remaining 460 patients were enrolled in the final analysis (Figure 1B). The cohort comprised 276 males (60.00%) and 184 females (40.00%). A total of 50 patients underwent molecular screening and received targeted therapy. Regarding clinical staging, 192 patients (41.74%) were classified as stage Ia, while 59 (12.83%), 53 (11.52%), 84 (18.26%), 54 (11.74%), and 18 (3.91%) were classified as stages Ib, IIa, IIb, IIIa, and IIIb, respectively (Table 1; Supplementary Table 1). The median follow-up duration was 31 months (range: 12–96 months). During the follow-up period, 94 patients (20.43%) experienced recurrence or metastasis after NSCLC resection, and 80 patients (17.39%) died.

**Table 1:** Baseline characteristics of patients with NSCLC.

Characteristics	Number	Percentage (%)
Patients’ characteristics		
Age (years)		
<50	48	10.43
≥50	412	89.57
Sex		
Male	276	60.00%
Female	184	40.00%
Smoking		
No	278	60.43%
Yes	182	39.57%
Medications		
Glucocorticoids		
No	129	28.04%
Yes	331	71.96%
Antibacterials		
No	30	6.52%
Yes	430	93.48%
Bronchodilator		
No	76	16.52%
Yes	384	83.48%
Mucolytic drugs		
No	49	10.65%
Yes	411	89.35%
Postoperative treatments		
Chemotherapy		
No	263	57.17%
Yes	197	42.83%
Targeted therapy		
No	409	88.91%
Yes	51	11.09%
Radiotherapy		
No	435	94.57%
Yes	25	5.43%
Perioperative conditions		
Blood loss		
<200 mL	313	68.04%
≥200 mL	147	31.96%

NSCLC, non-small cell lung cancer.

### 3.2 Optimal cut-off value for inflammatory markers

**Figure 2:**
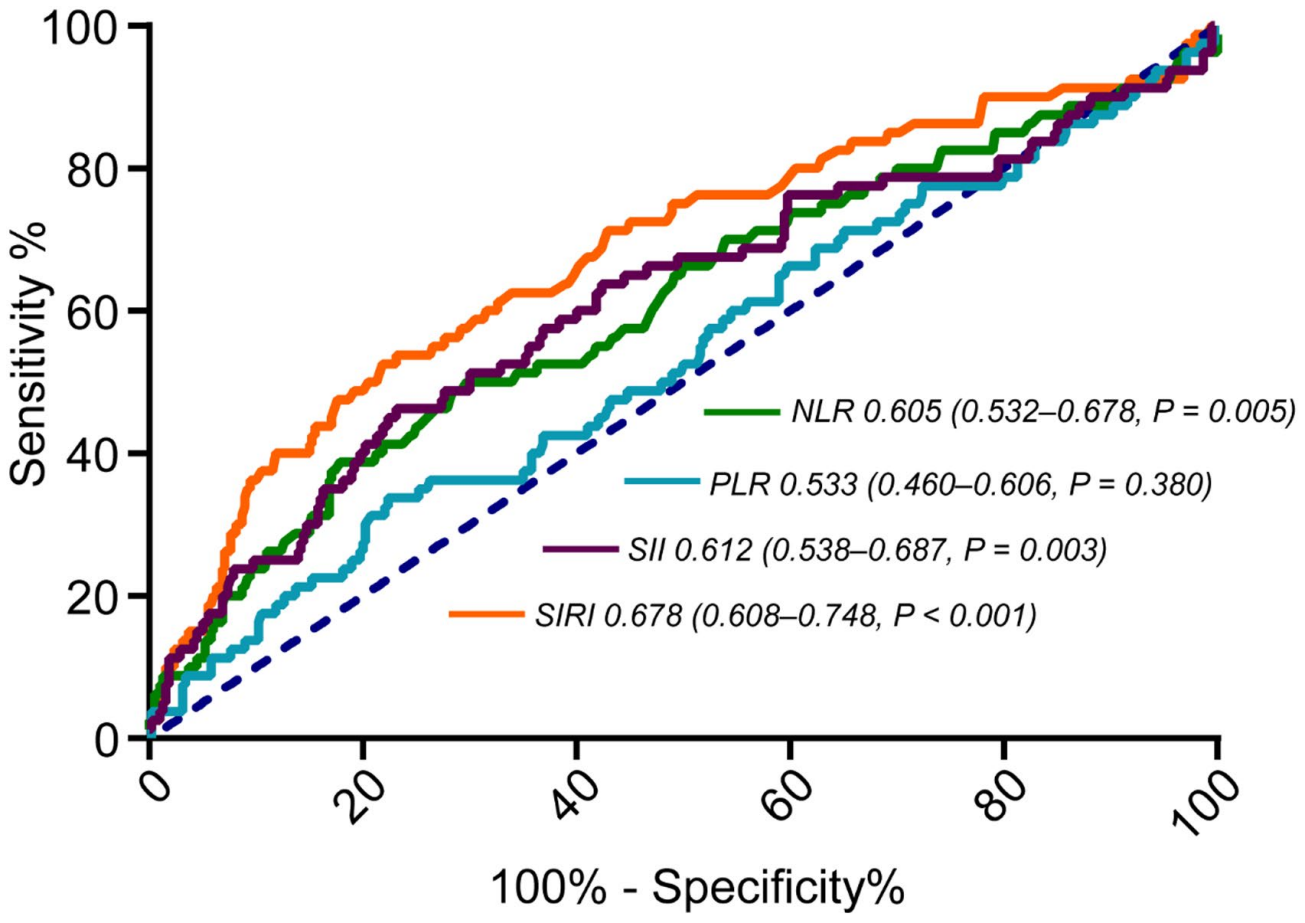
ROC curves showing optimal cut-off values for NLR, PLR, SII, and SIRI. ROC, receiver operating characteristics; NLR, neutrophil-to-lymphocyte ratio; PLR, platelet-to-lymphocyte ratio; SII, systemic immune-inflammation index; SIRI, systemic inflammation response index.

Based on the ROC curves, the optimal cut-off values for the inflammatory markers NLR, PLR, SII, and SIRI were determined. The AUC for PLR was 0.533 (95% CI: 0.460–0.606, *p* = 0.380), indicating limited predictive value. In contrast, the AUCs for NLR, SII, and SIRI were 0.605 (95% CI: 0.532–0.678, *p* = 0.005), 0.612 (95% CI: 0.538–0.687, *p* = 0.003), and 0.678 (95% CI: 0.608–0.748, *p* < 0.001), respectively, suggesting better prognostic utility (Figure 2). Patients were subsequently stratified into high and low groups based on the optimal cut-off values of pretreatment SIRI and NLR. Similar grouping was applied to all inflammatory markers to facilitate comparison between low and high inflammation subgroups (Tables 2, 3, Supplementary Tables 2, 3).

**Table 2:** Baseline characteristics according to NLR and PLR.

Characteristics	NLR < 3.47 (*n* = 158)	NLR ≥ 3.47 (*n* = 302)	*p*	PLR < 186 (*n* = 347)	PLR ≥ 186 (*n* = 113)	*p*
Age (years)						
<50	10 (6.33)	38 (12.58)	0.037*	36 (10.37)	12 (10.62)	0.941
≥50	148 (93.67)	264 (87.42)		311 (89.63)	101 (89.38)	
Sex						
Male	120 (75.95)	156 (51.66)	0.000***	206 (59.37)	70 (61.95)	0.627
Female	38 (24.05)	146 (48.34)		141 (40.63)	43 (38.05)	
Smoking						
No	82 (51.90)	196 (64.90)	0.007**	208 (59.94)	70 (61.95)	0.705
Yes	76 (48.10)	106 (35.10)		139 (40.06)	43 (38.05)	
TNM stage						
Ia	53 (33.54)	139 (46.03)	0.006**	151 (43.52)	41 (36.28)	0.455
Ib	19 (12.03)	40 (13.25)		47 (13.54)	12 (10.62)	
IIa	23 (14.56)	30 (9.93)		39 (11.24)	14 (12.39)	
IIb	37 (23.42)	47 (15.56)		57 (16.43)	27 (23.89)	
IIIa	15 (9.49)	39 (12.91)		39 (11.24)	15 (13.27)	
IIIb	11 (6.96)	7 (2.32)		14 (4.03)	4 (3.54)	
Preoperative white blood cell count						
<3.5109/L	2 (1.27)	2 (0.66)	0.000***	3 (0.86)	1 (0.88)	0.771
3.5–9.5109/L	106 (67.09)	259 (85.76)		278 (80.12)	87 (76.99)	
>9.5109/L	50 (31.65)	41 (13.58)		66 (19.02)	25 (22.12)	
Preoperative albumin level						
<40 g/dL	79 (50.00)	80 (26.49)	0.000***	113 (32.56)	46 (40.71)	0.251
40–55 g/dL	78 (49.37)	222 (73.51)		233 (67.15)	67 (59.29)	
>55 g/dL	1 (0.63)	0 (0.00)		1 (0.29)	0 (0.00)	
Operation time						
<3 h	30 (18.99)	88 (29.14)	0.018*	90 (25.94)	28 (24.78)	0.807
≥3 h	128 (81.01)	214 (70.86)		257 (74.06)	85 (75.22)	
Blood loss						
<200 mL	92 (58.23)	221 (73.18)	0.001**	241 (69.45)	72 (63.72)	0.256
≥200 mL	66 (41.77)	81 (26.82)		106 (30.55)	41 (36.28)	
Hospital stay						
<15 days	32 (20.25)	97 (32.12)	0.007**	100 (28.82)	29 (25.66)	0.517
≥15 days	126 (79.75)	205 (67.88)		247 (71.18)	84 (74.34)	

NSCLC, non-small cell lung cancer; NLR, neutrophil-to-lymphocyte ratio; PLR, platelet-to-lymphocyte ratio. *p < 0.05; **p < 0.01; ***p < 0.001.

**Table 3:** Baseline characteristics according to SII and SIRI.

Characteristics	SII < 853.71 (*n* = 334)	SII ≥ 853.71 (*n* = 126)	*p*	SIRI<1.66 (*n* = 334)	SIRI≥1.66 (*n* = 126)	*p*
Age (years)						
<50	35 (10.48)	13 (10.32)	0.96	39 (11.68)	9 (7.14)	0.156
≥50	299 (89.52)	113 (89.68)		295 (88.32)	117 (92.86)	
Sex						
Male	188 (56.29)	88 (69.84)	0.008**	179 (53.59)	97 (76.98)	0.000***
Female	146 (43.71)	38 (30.16)		155 (46.41)	29 (23.02)	
Smoking						
No	207 (61.98)	71 (56.35)	0.271	214 (64.07)	64 (50.79)	0.009***
Yes	127 (38.02)	55 (43.65)		120 (35.93)	62 (49.21)	
TNM stage						
Ia	154 (46.11)	38 (30.16)	0.041*	157 (47.01)	35 (27.78)	0.001**
Ib	42 (12.57)	17 (13.49)		44 (13.17)	15 (11.90)	
IIa	37 (11.08)	16 (12.70)		32 (9.58)	21 (16.67)	
IIb	55 (16.47)	29 (23.02)		53 (15.87)	31 (24.60)	
IIIa	36 (10.78)	18 (14.29)		39 (11.68)	15 (11.90)	
IIIb	10 (2.99)	8 (6.35)		9 (2.69)	9 (7.14)	
Preoperative white blood cell count						
<3.5109/L	4 (1.20)	0 (0.00)	0.000***	4 (1.20)	0 (0.00)	0.000***
3.5–9.5109/L	288 (86.23)	77 (61.11)		294 (88.02)	71 (56.35)	
>9.5109/L	42 (12.57)	49 (38.89)		36 (10.78)	55 (43.65)	
Preoperative albumin level						
<40 g/dL	103 (30.84)	56 (44.44)	0.021*	94 (28.14)	65 (51.59)	0.000***
40–55 g/dL	230 (68.86)	70 (55.56)		240 (71.86)	60 (47.63)	
>55 g/dL	1 (0.30)	0 (0.00)		0 (0.00)	1 (0.79)	
Operation time						
<3 h	84 (25.15)	34 (26.98)	0.688	92 (27.54)	26 (20.63)	0.13
≥3 h	250 (74.85)	92 (73.02)		242 (72.46)	100 (79.37)	
Blood loss						
<200 mL	241 (72.16)	72 (57.14)	0.002**	246 (73.65)	67 (53.17)	0.000***
≥200 mL	93 (27.84)	54 (42.86)		88 (26.35)	59 (46.83)	
Hospital stay						
<15 days	101 (30.24)	28 (22.22)	0.088	104 (31.14)	25 (19.84)	0.016*
≥15 days	233 (69.76)	98 (77.78)		230 (68.86)	101 (80.16)	

SII, systemic immune-inflammation index; SIRI, systemic inflammation response index. *p < 0.05; **p < 0.01; ***p < 0.001.

### 3.3 Survival analysis

**Figure 3:**
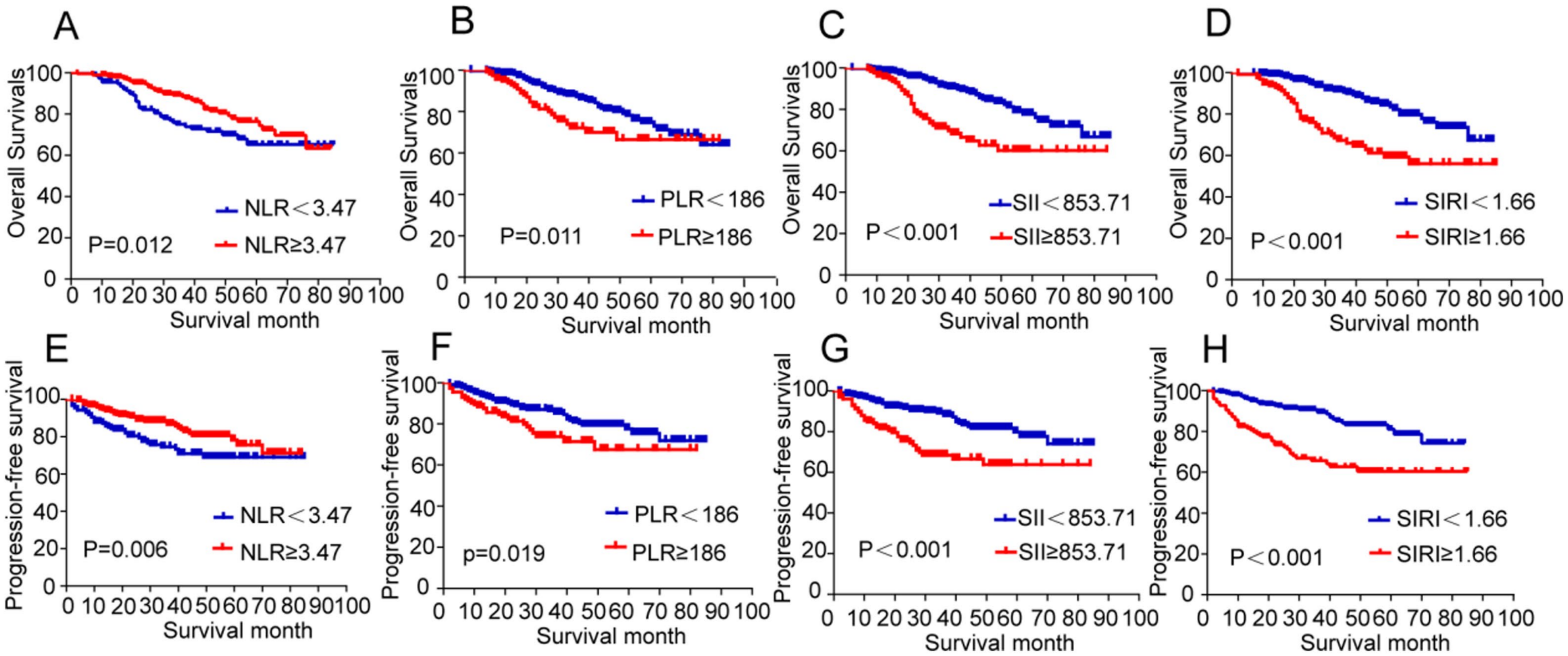
Kaplan–Meier survival curves showing overall survival rates and progression-free survival rates of patients undergoing primary resection for NSCLC. **(A–D)** Overall survival rates according to NLR, PLR, SII, and SIRI. **(E–H)** Progression-free survival rates according to NLR, PLR, SII, and SIRI. NSCLC, non-small cell lung cancer; NLR, neutrophil-to-lymphocyte ratio; PLR, platelet-to-lymphocyte ratio; SII, systemic immune-inflammation index; SIRI, systemic inflammation response index.

According to Kaplan–Meier survival curves, patients with low PLR, SII, and SIRI levels demonstrated significantly better OS compared to those in the high-level groups (Figures 3A–D). Similarly, patients with lower PLR, SII, and SIRI values exhibited superior PFS than their high-level counterparts (Figures 3E–H). The 1-, 3-, and 5-year OS rates for all resectable NSCLC patients included in the study were 97.4, 83.6, and 72.6%, respectively, while the corresponding PFS rates were 92.5, 82.4, and 75.0% (Tables 4, 5).

**Table 4:** 1-, 3-, and 5-year OS for patients with NSCLC.

OS	1-Year	3-Year	5-Year	*p*
All	97.4%	83.6%	72.6%	
NLR < 3.47 vs. NLR ≥ 3.47	95.6% vs. 98.3%	75.0% vs. 89.2%	65.3% vs. 76.5%	0.012*
PLR < 186 vs. PLR ≥ 186	98.3% vs. 94.7%	87.3% vs. 72.1%	74.7% vs. 66.5%	0.011*
SII < 853.71 vs. SII ≥ 853.71	98.2% vs. 95.2%	89.8% vs. 68.2%	77.7%vs. 60.15	*p* < 0.001***
SIRI<1.66 vs. SIRI≥1.66	98.8% vs. 93.6%	91.2% vs. 67.3%	79.7% vs. 56.0%	*p* < 0.001***

NSCLC, non-small cell lung cancer; NLR, neutrophil-to-lymphocyte ratio; PLR, platelet-to-lymphocyte ratio; SII, systemic immune-inflammation index; SIRI, systemic inflammation response index. *p < 0.05; **p < 0.01; ***p < 0.001.

**Table 5:** 1-, 3-, and 5-year PFS for patients with NSCLC.

PFS	1-Year	3-Year	5-Year	*p*
All	92.5%	82.4%	75.0%	
NLR < 3.47 vs. NLR ≥ 3.47	87.9% vs. 94.9%	74.6% vs. 88.3%	69.1% vs. 78.3%	0.006**
PLR < 186 vs. PLR ≥ 186	93.9% vs. 88.4%	85.3% vs. 73.9%	79.4% vs. 67.5%	0.019*
SII < 853.71vs SII ≥ 853.71	95.4% vs. 84.8%	88.0% vs. 68.5%	81.7% vs. 63.8%	*p* < 0.001***
SIRI<1.66 vs. SIRI≥1.66	96.3% vs. 82.5%	90.7% vs. 65.7%	81.1% vs. 60.5%	*p* < 0.001***

NSCLC, non-small cell lung cancer; NLR, neutrophil-to-lymphocyte ratio; PLR, platelet-to-lymphocyte ratio; SII, systemic immune-inflammation index; SIRI, systemic inflammation response index. *p < 0.05; **p < 0.01; ***p < 0.001.

### 3.4 Identification of significant prognostic factors

Univariate logistic analysis revealed female patients were at an increased risk for NLR and SIRI with a relative risk of 2.955 and 0.345. Also, smoking occurrence increased the risk of NLR and SIRI (Supplementary Tables 4, 5). Cox regression analysis was conducted to identify prognostic factors for NSCLC after primary surgical resection. Univariate analysis revealed that elevated NLR (HR = 0.546, 95% CI: 0.352–0.847, *p* = 0.007), PLR (HR = 1.728, 95% CI: 1.086–2.750, *p* = 0.021), SII (HR = 2.594, 95% CI: 1.669–4.032, *p* < 0.001), and SIRI (HR = 3.135, 95% CI: 2.021–4.864, *p* < 0.001) were significantly associated with poorer progression-free survival (PFS) (Table 6). Conversely, lower levels of NLR (HR = 0.572, 95% CI: 0.369–0.888, *p* = 0.013), PLR (HR = 1.816, 95% CI: 0.141–2.890, *p* = 0.012), SII (HR = 2.716, 95% CI: 1.746–4.224, *p* < 0.001), and SIRI (HR = 3.027, 95% CI: 1.950–4.697, *p* < 0.001) were significantly associated with improved OS. Additionally, TNM stage and chemotherapy were significantly associated with PFS, although they were not identified as independent prognostic factors for OS. Smoking status, lymphatic metastasis, antibacterial use, radiotherapy, intraoperative blood loss, preoperative white blood cell count and preoperative serum albumin were significantly associated with both OS and PFS.

**Table 6:** Univariate Cox analysis of PFS and OS.

Variable	PFS		OS	
	HR (95% CI)	*p*	HR (95% CI)	*p*
Smoking				
No	Reference	0.012*	Reference	0.046*
Yes	1.765 (1.135–2.746)		1.568 (1.008–2.439)	
TNM stage				
Ia	Reference	<0.001***	Reference	0.659
Ib	0.766 (0.254–2.308)		0.735 (0.224–2.214)	
IIa	3.503 (1.690–7.260)		2.976 (1.435–6.175)	
IIb	2.941 (1.493–5.791)		2.654 (1.346–5.230)	
IIIa	5.290 (2.685–10.421)		4.545 (2.307–8.952)	
IIIb	6.871 (3.004–15.717)		6.163 (2.696–14.091)	
Lymphatic metastasis				
No	Reference	<0.001***	Reference	<0.001***
Yes	4.579 (2.945–7.119)		4.242 (2.726–6.599)	
Chemotherapy				
No	Reference	0.013*	Reference	0.081
Yes	1.755 (1.127–2.733)		1.484 (0.952–2.312)	
Radiotherapy				
No	Reference	<0.001***	Reference	0.006**
Yes	3.312 (1.752–6.260)		2.442 (1.292–4.618)	
Antibacterials				
No	Reference	0.018*	Reference	0.042*
Yes	0.462 (0.244–0.875)		0.515 (0.272–0.975)	
Blood loss				
<200 mL	References	0.001**	References	0.008**
≥200 mL	2.181 (1.401–3.396)		1.831 (1.175–2.854)	
Preoperative white blood cell count				
<3.5109/L	1.666 (0.230–12.053)	0.007**	1.701 (0.235–12.316)	0.014*
3.5–9.5109/L	Reference		Reference	
>9.5109/L	1.921 (1.195–3.086)		1.809 (1.126–2.907)	
Preoperative albumin				
<40 g/dL	1.585 (1.019–2.466)	0.041*	1.603 (1.03–2.493)	0.044*
40–55 g/dL	Reference		Reference	
>55 g/dL	-		-	
NLR				
<3.470	Reference	0.007**	Reference	0.013*
≥3.470	0.546 (0.352–0.847)		0.572 (0.369–0.888)	
PLR				
<186	Reference	0.021*	Reference	0.012*
≥186	1.728 (1.086–2.750)		1.816 (0.141–2.890)	
SII				
<853.71	Reference	<0.001***	Reference	<0.001***
≥853.71	2.594 (1.669–4.032)		2.716 (1.746–4.224)	
SIRI				
<1.66	Reference	<0.001***	Reference	<0.001***
≥1.66	3.135 (2.021–4.864)		3.027 (1.950–4.697)	

NLR, neutrophil-to-lymphocyte ratio; PLR, platelet-to-lymphocyte ratio; SII, systemic immune-inflammation index; SIRI, systemic inflammation response index; OS, overall survival; PFS, progression-free survival. *p < 0.05; **p < 0.01; ***p < 0.001.

To identify independent prognostic factors, statistically significant variables from the univariate analysis—including smoking, chemotherapy, radiotherapy, intraoperative blood loss, preoperative white blood cell count, albumin level, as well as NLR, PLR, SII, and SIRI—were incorporated into a multivariate Cox regression model. For PFS, the results showed that lymphatic metastasis (HR = 3.101, 95% CI: 1.636–5.877, *p* = 0.001), radiotherapy (HR = 2.076, 95% CI: 1.052–4.099, *p* = 0.035), antibacterial use (HR = 0.425, 95% CI: 0.213–0.850, *p* = 0.016), NLR ≥ 3.470 (HR = 2.215, 95% CI: 1.054–4.657, *p* = 0.036), and SIRI ≥ 1.66 (HR = 3.612, 95% CI: 1.548–8.426, *p* = 0.003) were identified as independent prognostic factors. For overall survival (OS), lymphatic metastasis (HR = 4.118, 95% CI: 2.608–6.501, *p* < 0.001), antibacterial use (HR = 0.435, 95% CI: 0.220–0.863, *p* = 0.017), NLR ≥ 3.470 (HR = 2.158, 95% CI: 1.045–4.457, *p* = 0.038), and SIRI ≥ 1.66 (HR = 3.442, 95% CI: 1.492–7.938, *p* = 0.004) were independently associated with prognosis (Table 7).

**Table 7:** Multivariate Cox analysis of PFS and OS.

Variable	PFS		OS	
	HR (95% CI)	*p*	HR (95% CI)	*p*
Lymphatic metastasis				
No	Reference	0.001**	Reference	<0.001***
Yes	3.101 (1.636–5.877)		4.118 (2.608–6.501)	
Radiotherapy				
No	Reference	0.035*	Reference	0.221
Yes	2.076 (1.052–4.099)		1.525 (0.776–2.996)	
Antibacterials				
No	Reference	0.016*	Reference	0.017*
Yes	0.425 (0.213–0.850)		0.435 (0.220–0.863)	
NLR				
<3.470	Reference	0.036*	Reference	0.038*
≥3.470	2.215 (1.054–4.657)		2.158 (1.045–4.457)	
SIRI				
<1.66	Reference	0.003**	Reference	0.004**
≥1.66	3.612 (1.548–8.426)		3.442 (1.492–7.938)	

NLR, neutrophil-to-lymphocyte ratio; PLR, platelet-to-lymphocyte ratio; SII, systemic immune-inflammation index; SIRI, systemic inflammation response index; OS, overall survival; PFS, progression-free survival. *p < 0.05; **p < 0.01; ***p < 0.001.

### 3.5 Integrated analysis of consistent prognostic factors across multiple models

To enhance the reliability of prognostic factor selection, we incorporated 20 independent variables identified through univariate Cox analysis to model two dependent outcomes: PFS and OS. Three machine learning algorithms—LASSO regression, SVM, and RF—were employed for feature selection and model development. Despite differences in methodological approaches, several variables consistently demonstrated strong associations with both OS and PFS across all three models.

**Figure 4:**
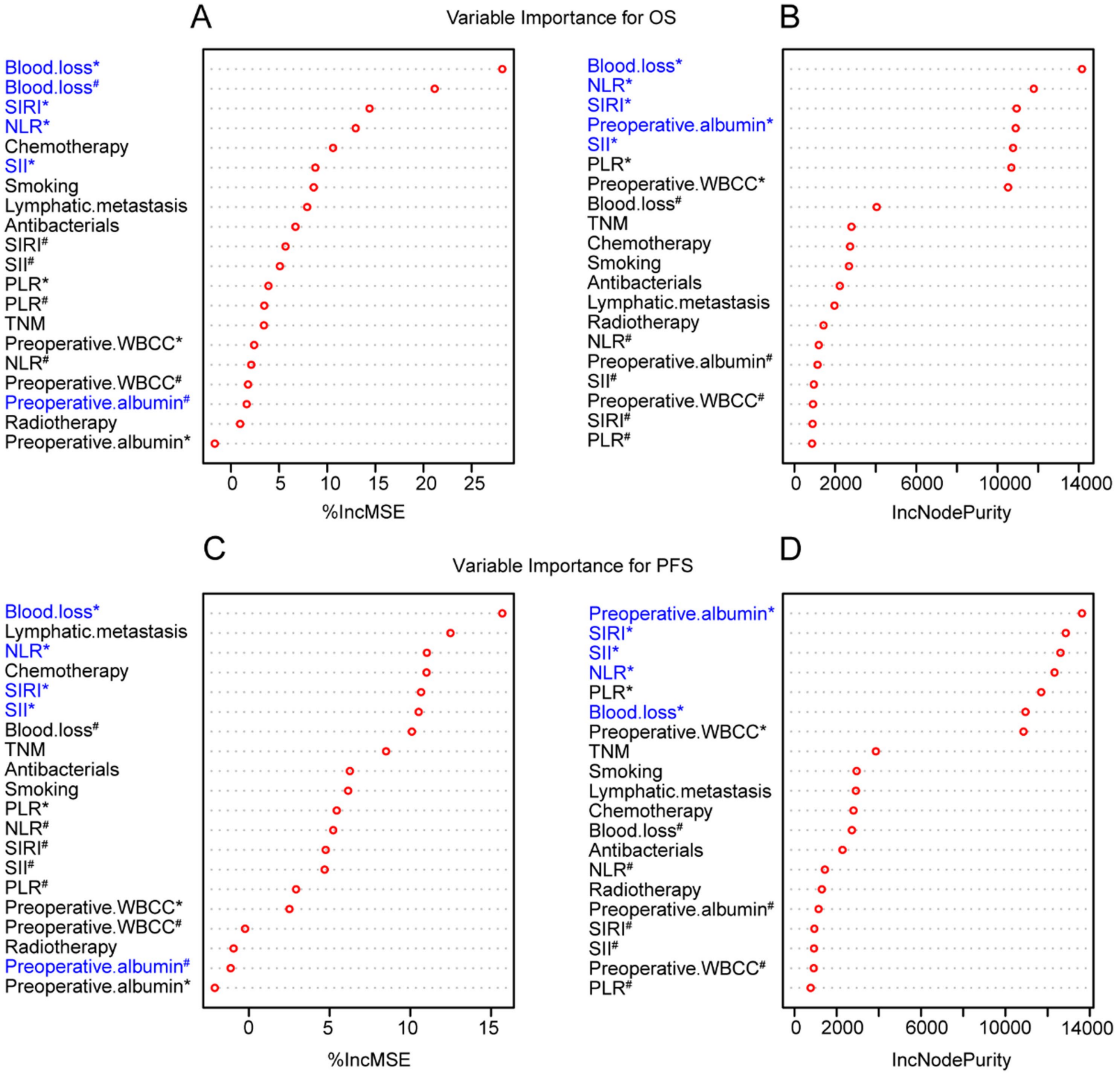
Random forest models showing important variables for overall survival rates and progression-free survival rates of patients undergoing primary resection for NSCLC. **(A,B)** %IncMSE and IncNodePurity of random forest models for OS. **(C,D)** %IncMSE and IncNodePurity of random forest models for PFS. ^*^Continuous variable; ^#^Categorical variable. NSCLC, non-small cell lung cancer; NLR, neutrophil-to-lymphocyte ratio; PLR, platelet-to-lymphocyte ratio; SII, systemic immune-inflammation index; SIRI, systemic inflammation response index; WBCC, white blood cell count.

**Figure 5:**
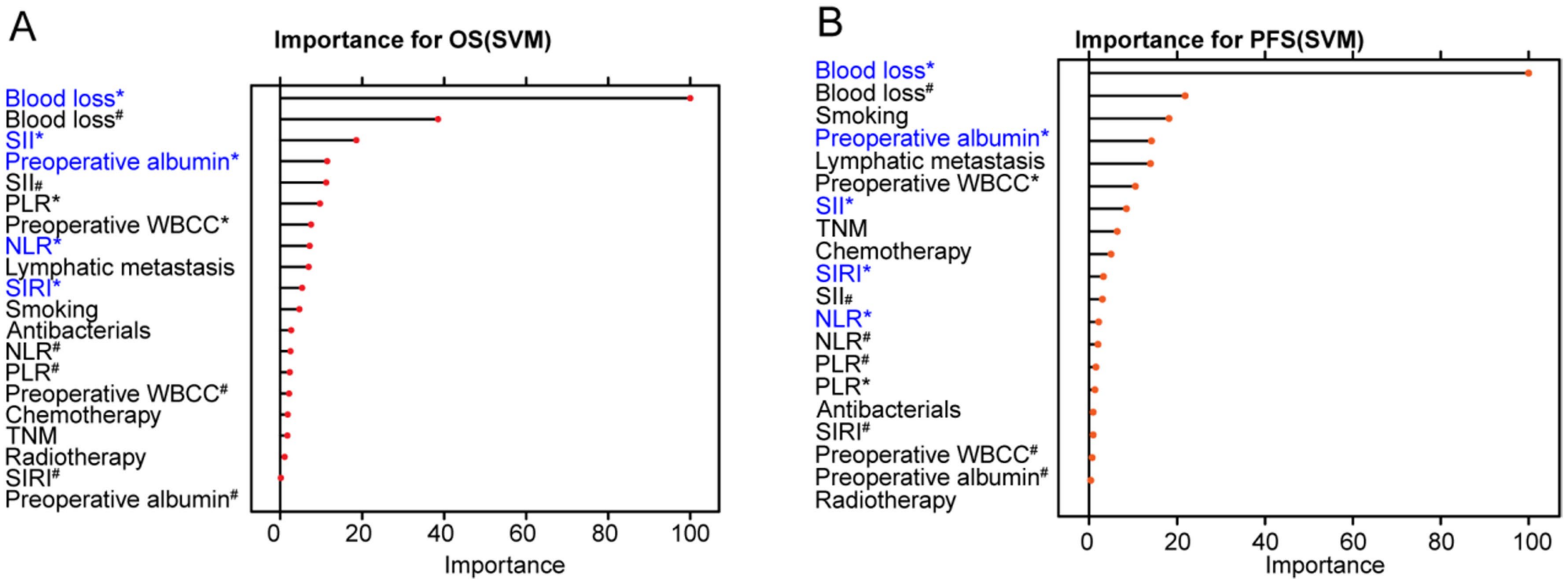
Support Vector Machine models showing important variables for overall survival rates and progression-free survival rates of patients undergoing primary resection for NSCLC. **(A)** Importance of Support Vector Machine models for OS. **(B)** Importance of Support Vector Machine models for PFS. *Continuous variable; ^#^Categorical variable. NSCLC, non-small cell lung cancer; NLR, neutrophil-to-lymphocyte ratio; PLR, platelet-to-lymphocyte ratio; SII, systemic immune-inflammation index; SIRI, systemic inflammation response index; WBCC, white blood cell count.

Among the inflammation-related markers, SII was consistently identified as a top predictor across all three machine learning models. SIRI and NLR also emerged as key predictors, particularly in the SVM and RF models. Additionally, perioperative blood loss and preoperative albumin levels were selected by all three approaches. In the RF model, several variables—most notably perioperative blood loss, NLR, and SIRI—showed the highest contribution to the prediction accuracy for PFS (Figures 4A–D). The SVM model similarly confirmed SIRI, SII, NLR, perioperative blood loss, and preoperative albumin as the leading predictors of PFS (Figures 5A,B). In the LASSO model, smoking, lymphatic metastasis, chemotherapy, perioperative blood loss, preoperative albumin, and SII were retained at the optimal penalty value for OS, while smoking, lymphatic metastasis perioperative blood loss, preoperative albumin, and SII were kept for PFS (Supplementary Figures 1A–D). These findings demonstrate a high degree of consistency across both statistical and machine learning approaches, underscoring the prognostic importance of systemic inflammatory markers and perioperative clinical factors.

### 3.6 Nomogram model constructed based on risk factors for predicting postoperative outcome

**Figure 6:**
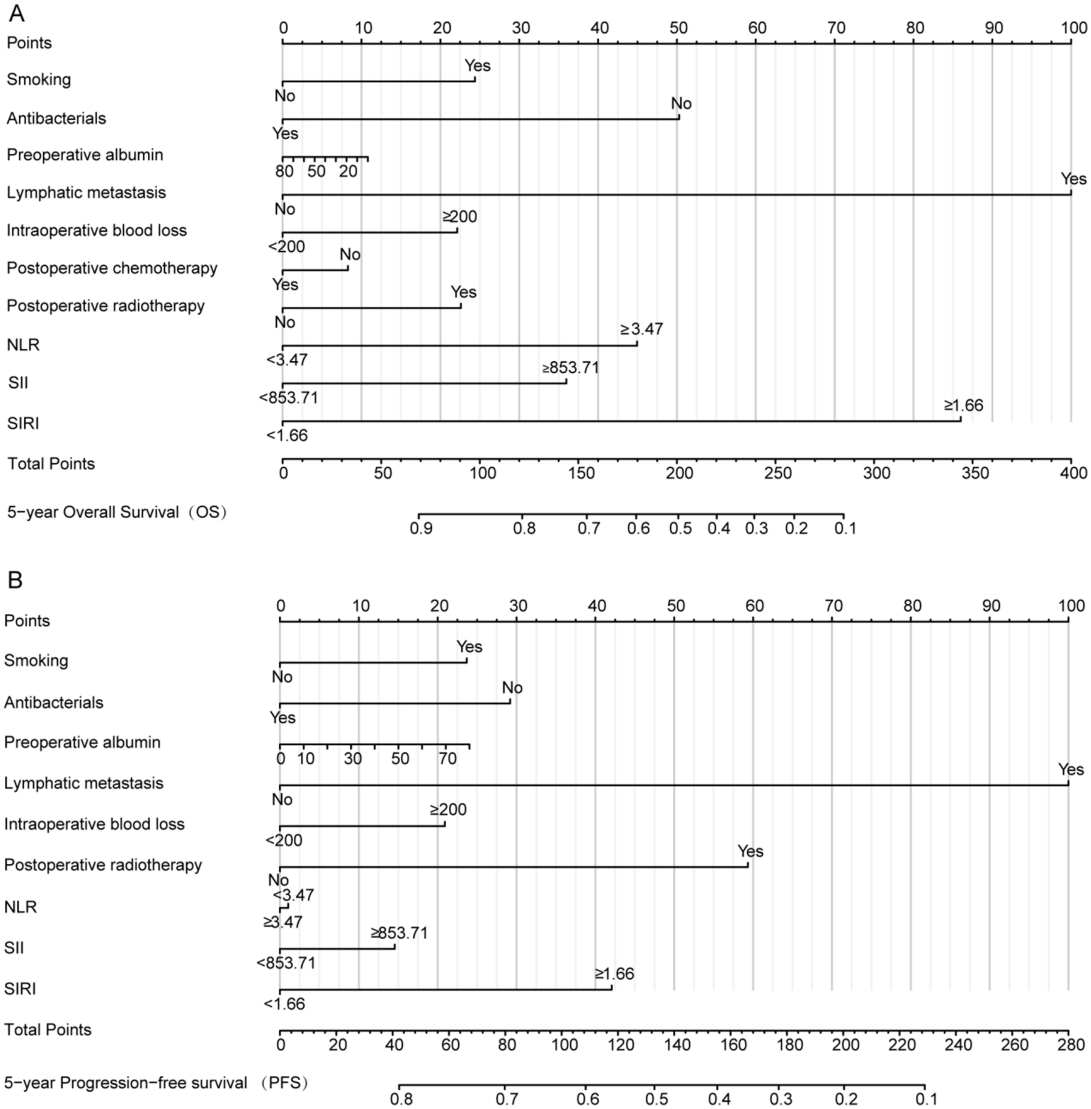
Nomogram was used for predicting postoperative outcome. **(A)** OS; **(B)** PFS. NLR, neutrophil-to-lymphocyte ratio; SII, systemic immune-inflammation index; SIRI, systemic inflammation response index.

Based on the independent predictors identified through multivariate Cox regression analysis and three machine learning algorithms—LASSO regression, SVM, and RF—used in this study, a nomogram was developed (Figure 6). The nomogram was developed to predict two postoperative outcomes: 5-year PFS and 5-year OS. Each level of all variables was assigned a corresponding score on the scale. The probability of recurrence or death for each patient was calculated by summing the scores assigned to each predictor variable. Patients with higher nomogram scores had a higher risk of recurrence or death.

### 3.7 External validation of significant prognostic factors

**Figure 7:**
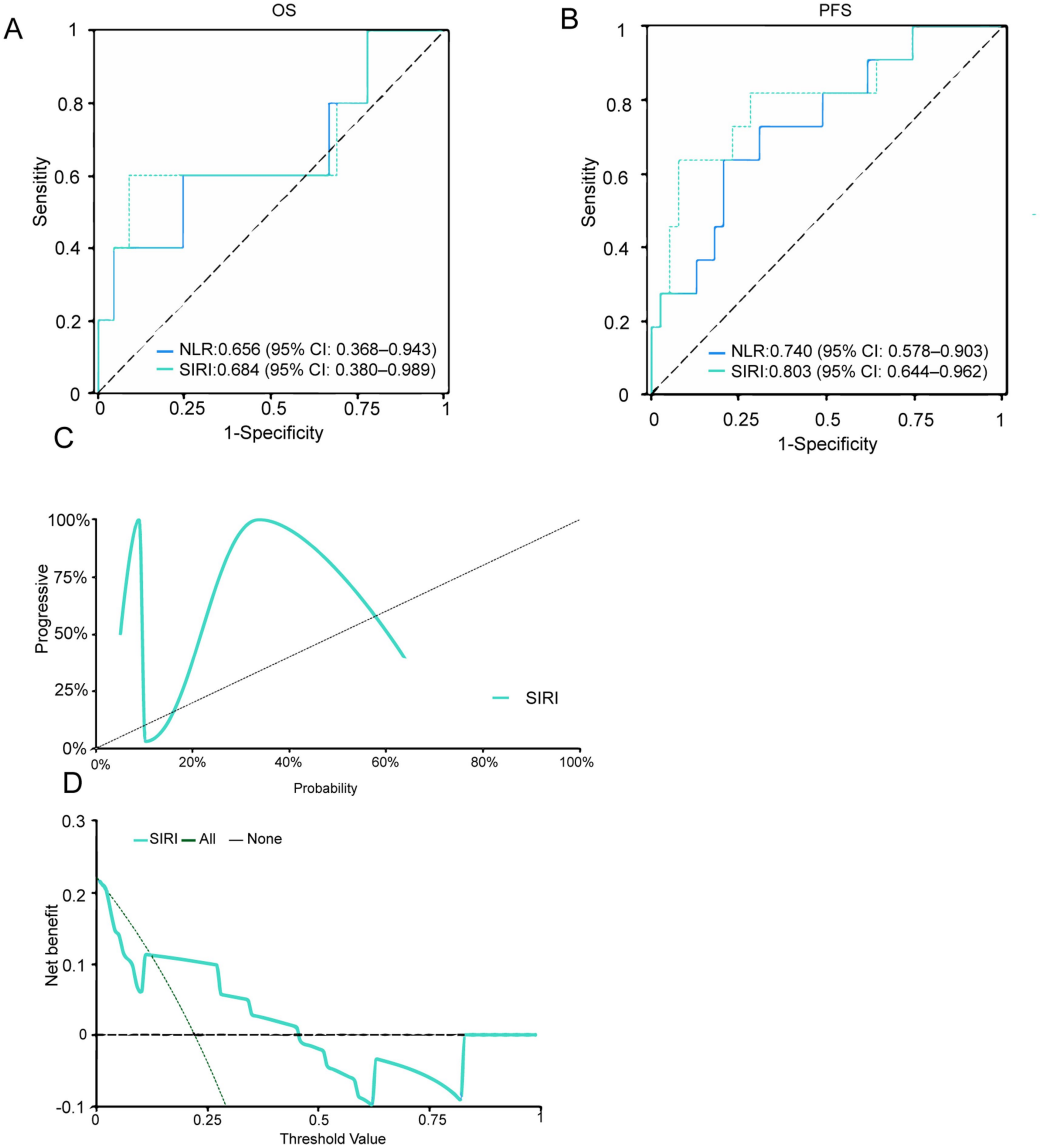
**(A,B)** ROC curves. **(C)** Calibration curve. **(D)** Decision curve analysis (DCA). NLR, neutrophil-to-lymphocyte ratio; SIRI, systemic inflammation response index.

The comparison of significant variable data for the external independent patient cohort has shown (Supplementary Tables 6, 7). For OS, the C-index values for NLR and SIRI in external validation were 0.656 (95% CI: 0.368–0.943) and 0.684 (95% CI: 0.380–0.989), respectively. For PFS, the C-index values were 0.740 (95% CI: 0.578–0.903) and 0.803 (95% CI: 0.644–0.962), indicating that both NLR and SIRI exhibit good discriminative performance and meaningful predictive ability for PFS (Figures 7A,B). The calibration curve demonstrates strong agreement between observed and predicted outcomes suggesting good consistency (Figure 7C). Decision curve analysis (DCA) for external validation indicates that SIRI provides a higher net benefit in predicting PFS across a range of clinically relevant threshold probabilities (Figure 7D).

## 4 Discussion

In this study, we retrospectively analyzed data from 460 NSCLC patients treated at two comprehensive hospitals in Guangdong Province to investigate the prognostic value of preoperative inflammatory markers—NLR, PLR, SII, and SIRI—in patients undergoing primary surgical resection. Our findings demonstrated that elevated levels of systemic inflammatory markers, particularly SIRI, were significantly associated with poorer overall survival (OS) and progression-free survival (PFS). These results are consistent with previous meta-analyses reporting that high pretreatment NLR is associated with shorter OS and PFS in NSCLC patients (20), and that elevated SIRI independently correlates with worse clinical outcomes in NSCLC (21).

A previous retrospective study by Wenliang Liu et al. (22) demonstrated that in patients with operable NSCLC who received neoadjuvant chemotherapy combined with immunotherapy, a higher preoperative NLR was associated with shorter disease-free survival (DFS). Mechanistically, an elevated NLR reflects a shift toward a pro-tumor inflammatory microenvironment: neutrophils secrete cytokines (e.g., IL-6, TNF-*α*), growth factors (e.g., VEGF), and proteolytic enzymes (e.g., MMPs), all of which promote angiogenesis, tumor proliferation, invasion, and metastasis. Conversely, lymphocytes are central mediators of antitumor immunity; a reduced lymphocyte count reflects impaired adaptive immune responses. The balance between neutrophil-driven tumor promotion and lymphocyte-mediated tumor control likely underpins the prognostic value of NLR for both OS and PFS (23). SIRI, which further incorporates monocyte counts, captures the contribution of monocyte-derived tumor-associated macrophages (TAMs) that support immunosuppression, angiogenesis, and metastatic progression. To date, research on SIRI in NSCLC has primarily focused on patients with advanced disease, including those treated with immune checkpoint inhibitors (ICIs) (24), EGFR-TKIs (25), PD-1 inhibitors (26), patients with unresectable stage IIIB/C NSCLC (27), and those with brain metastases (28). These studies have demonstrated that pre- and post-treatment SIRI levels serve as independent prognostic indicators in patients undergoing drug-based and adjunctive therapies, highlighting the clinical utility of SIRI in monitoring treatment response and disease progression. In contrast, our study focused on preoperative SIRI in patients with resectable NSCLC undergoing primary surgical treatment. Using a multicenter cohort of 460 patients, our findings revealed that a high preoperative SIRI was a robust independent predictor of poorer postoperative outcomes. Compared to NLR alone, SIRI provides a more comprehensive reflection of systemic inflammation and immune dysregulation, reinforcing its potential role in perioperative risk stratification for surgical NSCLC patients (21). It is noteworthy that female sex and smoking status were independently associated with elevated NLR and SIRI. However, previous studies reported smokers exhibited significantly higher NLR and PLR compared with non-smokers, contributing to elevated SIRI in cancer patients, which was consistent with our study (29). Tobacco smoke, a potent inflammatory irritant of the respiratory tract, can persistently activate neutrophils and monocytes and impairs lymphocyte function, (30) collectively driving smoking-associated elevation of SIRI.

In addition to inflammatory indices, lymphatic metastasis and radiotherapy were also closely associated with the prognosis of patients undergoing primary NSCLC resection. Lymphatic metastasis is universally recognized as a key mechanism of cancer dissemination. Tumor cells initially invade through the epithelial basement membrane, traverse the underlying connective tissue, and subsequently enter lymphatic vessels, ultimately colonizing and disrupting lymph nodes (31). Radiotherapy, while widely used as a local adjuvant treatment, has been reported to potentially accelerate metastasis by promoting tumor cell shedding into the lymphatic system. Lymph nodes, despite their immune activity, provide limited physical barriers to tumor cells. The nature and strength of immune responses within lymph nodes—particularly lymphocyte-mediated responses—may be critical in determining prognosis (32). This observation is consistent with our findings that emphasize the prognostic significance of inflammatory markers such as NLR and SIRI, both of which incorporate lymphocyte counts. Preoperative NLR and SIRI can serve as indirect indicators of baseline immune competence, which is closely linked to postoperative outcomes. An intact immune response facilitates recovery following NSCLC surgery and may enhance resistance to recurrence. Furthermore, our results also revealed that perioperative factors—such as intraoperative blood loss and preoperative serum albumin levels—were independently predictive of survival. These variables reflect surgical stress and the patient’s baseline nutritional and immunological status, both of which are known to influence recovery trajectories and long-term prognosis. This supports existing evidence suggesting that increased surgical burden and inadequate nutritional reserves may impair postoperative resilience and potentially facilitate progression of residual disease.

Although molecular minimal residual disease (MRD) detection using circulating tumor DNA (ctDNA) offers high specificity for early relapse—often identifying recurrence months before radiological evidence—it remains limited in widespread clinical application due to high cost, technical complexity, and inconsistent availability across institutions (33, 34). In contrast, inflammatory indices such as NLR, SIRI, and related markers are derived from routine complete blood counts, making them inexpensive, widely available, and capable of providing rapid results (35). Preoperative assessment of these markers offers a practical and low-cost tool for initial risk stratification. Patients exhibiting elevated NLR or SIRI levels may be prioritized for more intensive ctDNA-based MRD surveillance following surgery, thereby optimizing the allocation of healthcare resources. High-risk individuals—identified through both inflammatory markers and positive MRD—stand to benefit most from closer monitoring and timely adjuvant interventions (36, 37). These indices may guide perioperative immunotherapy, monitor preoperative therapy response, and inform risk-adapted surveillance—including more frequent follow-up and targeted use of additional monitoring tools.

In particular, this study aims to frame NLR/PLR/SII/SIRI as low-cost, readily available adjunct risk markers that may help inform risk-adapted surveillance intensity in settings where additional tests (including MRD) are not universally available, rather than as a stand-alone determinant for MRD testing. Integrating inflammatory markers with MRD assays in predictive models may further enhance prognostic accuracy. Importantly, such integration may inform risk-stratified therapeutic decisions, including the selection and timing of adjuvant immunotherapy. Emerging evidence from studies in solid tumors suggests that multi-modal algorithms outperform single-modality approaches in risk stratification (38, 39). In the context of NSCLC, combining preoperative inflammatory markers such as NLR or SIRI with postoperative ctDNA-based MRD status could refine postoperative management strategies. For instance, among patients with similar TNM stages, those presenting with both elevated inflammatory indices and detectable ctDNA would be classified as high-risk and may benefit from intensified adjuvant therapy or enrollment in clinical trials.

Although the results derived from traditional logistic regression and machine learning models were not entirely consistent, our multi-model analysis consistently identified NLR and SIRI as top prognostic predictors. This convergence reinforces the robustness of these markers and reduces potential model-specific biases. Given their low cost and widespread availability, NLR and SIRI are readily translatable into clinical practice, particularly in resource-limited settings where access to advanced molecular assays such as ctDNA is constrained. Collectively, our results suggest that these two variables may serve as valuable tools for survival risk stratification in patients with resectable NSCLC.

## 5 Limitation

Our study has demonstrated the highlighted NLR and SIRI as reliable independent prognostic indicators in patients with resectable NSCLC. However, future prospective studies are still needed to validate the optimal cut-off values for NLR and SIRI and conduct cost-effectiveness analyses to support their incorporation into personalized NSCLC management strategies. Moreover, given the limited sample size and pre-existing imbalances across key clinical subgroups, our cohort is underpowered to detect clinically meaningful differences in inflammation-based index associations, thereby precluding definitive subgroup analyses. Interventional trials should explore whether modulation of systemic inflammation—such as through perioperative anti-inflammatory interventions—can improve outcomes in patients with elevated preoperative NLR or SIRI. It is worth noting that, in the machine learning aspect of data analysis, NLR, PLR, SII, and SIRI appear to be more valuable as continuous variables than as categorical ones. However, Ash Kieran Clift et al. reported that regression-based methods exhibited better and more consistent performance than machine learning approaches when using clinical predictors available (40). The traditional logistic regression model remains a cornerstone statistical method in clinical and epidemiological research. Defining more precise categorical variables requires a larger sample size. Our next research direction is to compare traditional logistic regression with machine learning methods for developing more robust prognostic models in large-sample.

## 6 Conclusion

In conclusion, preoperative inflammatory indices—particularly NLR and SIRI—provide practical and cost-effective prognostic information. When integrated with TNM staging, MRD monitoring and immunotherapy molecular, these markers have the potential to enhance risk stratification and support personalized treatment strategies for patients with resectable NSCLC.

## Data Availability

The original contributions presented in the study are included in the article/Supplementary material, further inquiries can be directed to the corresponding authors.
