# Supplementary material for: Prognostic value of preoperative inflammatory markers in resectable non-small cell lung cancer: a multi-center retrospective study based on logistic regression and machine learning techniques
Source: Front Med (Lausanne). 2026 Apr 22;13:1771545. doi: 10.3389/fmed.2026.1771545 (PMC13143947; doi:10.3389/fmed.2026.1771545)
Supplement: Supplementary file 1 [file Data_Sheet_1.docx]

Supplemental Table 1 Baseline Characteristics of Patients with NSCLC

| **Characteristics** | **Number** | **Percentage (%)** |
| --- | --- | --- |
| **Preoperative comorbidities** |  |  |
| **Hypertension**  No  Yes | 374  86 | 81.30%  18.70% |
| **Diabetes mellitus**  No  Yes | 417  43 | 90.65%  9.35% |
| **Cerebrovascular disease**  No  Yes | 322  138 | 70.00%  30.00% |
| **Chronic obstructive disease**  No  Yes | 361  99 | 78.48%  21.52% |
| **Pulmonary atelectasis**  No  Yes | 442  18 | 96.09%  3.91% |
| **Lung surgery**  No  Yes | 455  5 | 98.91%  1.09% |
| **Perioperative conditions** |  |  |
| **Type of lung cancer surgery**  Central  Peripheral | 37  423 | 8.04%  91.96% |
| **Histological**  Adenocarcinoma  Squamous cell carcinoma  Other types | 368  90  2 | 80.00%  19.57%  0.43% |
| **TNM stage**  Ia  Ib  IIa  IIb  IIIa  IIIb | 192  59  53  84  54  18 | 41.74%  12.83%  11.52%  18.26%  11.74%  3.91% |
| **Lymphatic metastasis**  No  Yes | 349  111 | 75.87%  24.13% |
| **Hospital stay**  <15 days  ≥15 days | 129  331 | 28.04%  71.96% |
| ICU stay  <3 days  ≥3 days | 440  20 | 95.65%  4.35% |
| **Operation time**  <3 h  ≥3 h | 118  342 | 25.65%  74.35% |
| **Wound infection**  No  Yes | 459  1 | 99.78%  0.22% |
| **Reintubation**  No  Yes | 459  1 | 99.78%  0.22% |
| **Blood parameters** |  |  |
| **Preoperative white blood cell count**  <3.5 109/L  3.5–9.5 109/L  >9.5 109/L | 4  365  91 | 0.87%  79.35%  19.78% |
| **Preoperative albumin**  <40 g/dL  40–55 g/dL  >55 g/dL | 159  300  1 | 34.57%  65.22%  0.22% |

**Abbreviations:** NSCLC, non-small cell lung cancer

Supplemental Table 2 Baseline Characteristics According to NLR and PLR

| **Characteristics** | **NLR <3.47**  **(n=158)** | **NLR ≥3.47**  **(n=302)** | **P** | **PLR<186**  **(n=347)** | **PLR≥186**  **(n=113)** | **P** |
| --- | --- | --- | --- | --- | --- | --- |
| **Hypertension**  No  Yes | 116(73.42)  42(26.58) | 258(85.43)  44(14.57) | 0.002** | 290(83.57)  57(16.43) | 84(74.34)  29(25.66) | 0.029* |
| **Chronic obstructive disease**  No  Yes | 110(69.62)  48(30.38) | 251(83.11)  51(16.89) | 0.001** | 273(78.67)  74(21.33) | 88(77.88)  25(22.12) | 0.858 |
| **Histological**  Adenocarcinoma  Squamous cell carcinoma  Other types | 108(68.35)  49(31.01)  1(0.63) | 260(86.09)  41(13.58)  1(0.33) | 0.000*** | 285(82.13)  61(17.58)  1(0.29) | 83(73.45)  29(25.66)  1(0.88) | 0.114 |
| **Lymphatic metastasis**  No  Yes | 119(75.32)  39(24.68) | 230(76.16)  72(23.84) | 0.841 | 264(76.08)  83(23.92) | 85(75.22)  28(24.78) | 0.853 |
| **ICU stay**  <3 days  ≥3 days | 149(94.30)  9(5.70) | 291(96.36)  11(3.64) | 0.305 | 330(95.10)  17(4.90) | 110(97.35)  3(2.65) | 0.31 |
| **Antibacterials**  No  Yes | 14(8.86)  144(91.14) | 16(5.30)  286(94.70) | 0.142 | 24(6.92)  323(93.08) | 6(5.31)  107(94.69) | 0.548 |
| **Mucolytic drugs**  No  Yes | 23(14.56)  135(85.44) | 26(8.61)  276(91.39) | 0.050* | 36(10.37)  311(89.63) | 13(11.50)  100(88.50) | 0.735 |
| **Chemotherapy**  No  Yes | 85(53.80)  73(46.20) | 178(58.94)  124(41.06) | 0.29 | 200(57.64)  147(42.36) | 63(55.75)  50(44.25) | 0.725 |
| **Targeted therapy**  No  Yes | 143(90.51)  15(9.49) | 266(88.08)  36(11.92) | 0.431 | 310(89.34)  37(10.66) | 99(87.61)  14(12.39) | 0.612 |
| **Radiotherapy**  No  Yes | 143(90.51)  15(9.49) | 292(96.69)  10(3.31) | 0.005** | 328(94.52)  19(5.48) | 107(94.69)  6(5.31) | 0.946 |

**Abbreviations:** NLR, neutrophil-to-lymphocyte ratio; PLR, platelet-to-lymphocyte ratio. P<0.05*; P<0.01**; P<0.001***

Supplemental Table 3 Baseline Characteristics According to SII and SIRI

| Characteristics | SII <853.71  (n=334) | SII≥853.71  (n=126) | P | SIRI<1.66  (n=334) | SIRI≥1.66  (n=126) | P |
| --- | --- | --- | --- | --- | --- | --- |
| **Hypertension**  No  Yes | 279(83.53)  55(16.47) | 95(75.4)  31(24.6) | 0.046* | 280(83.83)  54(16.17) | 94(74.60)  32(25.40) | 0.024* |
| **Cerebrovascular disease**  No  Yes | 238(71.26)  96(28.74) | 84(66.67)  42(33.33) | 0.338 | 243(72.75)  91(27.25) | 79(62.70)  47(37.30) | 0.036* |
| **Chronic obstructive disease**  No  Yes | 267(79.94)  67(20.06) | 94(74.60)  32(25.40) | 0.214 | 276(82.63)  58(17.37) | 85(67.46)  41(32.54) | 0.000** |
| **Pulmonary atelectasis**  No  Yes | 323(96.71)  11(3.29) | 119(94.44)  7(5.56) | 0.264 | 325(97.31)  9(2.69) | 117(92.86)  9(7.14) | 0.028* |
| **Histological**  Adenocarcinoma  Squamous cell carcinoma  Other types | 285(85.33)  48(14.37)  1(0.30) | 83(65.87)  42(33.33)  1(0.79) | 0.000*** | 291(87.13)  42(12.57)  1(0.30) | 77(61.11)  48(38.10)  1(0.79) | 0.000*** |
| **Lymphatic metastasis**  No  Yes | 256(76.65)  78(23.35) | 93(73.81)  33(26.19) | 0.526 | 256(76.65)  78(23.35) | 93(73.81)  33(26.19) | 0.526 |
| **ICU stay**  <3 days  ≥3 days | 318(95.21)  16(4.79) | 122(96.83)  4(3.17) | 0.449 | 321(96.11)  13(3.89) | 119(94.44)  7(5.56) | 0.435 |
| **Antibacterials**  No  Yes | 21(6.29)  313(93.71) | 9(7.14)  117(92.86) | 0.74 | 17(5.09)  317(94.91) | 13(10.32)  113(89.68) | 0.043* |
| **Mucolytic drugs**  No  Yes | 30(8.98)  304(91.02) | 19(15.08)  107(84.92) | 0.059 | 24(7.19)  310(92.81) | 25(19.84)  101(80.16) | 0.000*** |
| **Chemotherapy**  No  Yes | 203(60.78)  131(39.22) | 60(47.62)  66(52.38) | 0.011* | 202(60.48)  132(39.52) | 61(48.41)  65(51.59) | 0.020* |
| **Targeted therapy**  No  Yes | 296(88.62)  38(11.38) | 113(89.68)  13(10.32) | 0.747 | 296(88.62)  38(11.38) | 113(89.68)  13(10.32) | 0.747 |
| **Radiotherapy**  No  Yes | 319(95.51)  15(4.49) | 116(92.06)  10(7.94) | 0.146 | 321(96.11)  13(3.89) | 114(90.48)  12(9.52) | 0.017* |

**Abbreviations:** SII, systemic immune-inflammation index; SIRI, systemic inflammation response index. P<0.05*; P<0.01**; P<0.001***

Supplemental Table 4 Univariate Cox Analysis of NLR and PLR

| Variable | NLR | | PLR | |
| --- | --- | --- | --- | --- |
|  | HR (95% CI) | P | HR (95% CI) | P |
| **Sex**  Male  Female | Reference  2.955(1.924-4.539) | P＜0.001  *** | Reference  0.897(0.580-1.388) | 0.627 |
| **Smoking**  No  Yes | Reference  0.584(0.394-0.863) | 0.007** | Reference  0.919(0.594-1.422) | 0.705 |

Supplemental Table 5 Univariate Cox Analysis of SII and SIRI

|  | SII | | SIRI | |
| --- | --- | --- | --- | --- |
|  | HR (95% CI) | P | HR (95% CI) | P |
| **Sex**  Male  Female | Reference  0.566(0.359-0.861) | 0.009** | Reference  0.345(0.216-0.551) | P＜0.001  *** |
| **Smoking**  No  Yes | Reference  1.263(0.833-1.913) | 0.272 | Reference  1.728(1.141-2.615) | 0.01* |

Supplemental Table 6 OS-relevant variable data for external patients

| **Variable** | **All patients in the external**  **cohort (N = 50)** | **Non-Survival**  **（N=45）** | **Survival**  **（N=5）** | **P Value** |
| --- | --- | --- | --- | --- |
| **Lymphatic metastasis**  No  Yes | 39（78.00%）  11（22.00%） | 36（80.00%）  9（20.00%） | 3（60.00%）  2（40.00%） | 0.64 |
| **Radiotherapy**  No  Yes | 48（96.00%）  2（4.00%） | 44（97.78%）  1（2.22%） | 4（80.00%）  1（20.00%） | 0.19 |
| **Antibacterials**  No  Yes | 1（2.00%）  49（98.00%） | 1（2.22%）  44（97.78%） | 0（0.00%）  5（100.00%） | 1 |
| **NLR**  <3.470  ≥3.470 | 41（82.00%）  9（18.00%） | 38（84.44%）  7（15.56%） | 3（60.00%）  2（40） | 0.21 |
| **SIRI**  <1.66  ≥1.66 | 40（80.00%）  10（20.00%） | 38（84.44%）  7（15.56%） | 2（40.00%）  3（60.00%） | 0.07 |

**Abbreviations:** NLR, neutrophil-to-lymphocyte ratio; SIRI, systemic inflammation response index; OS, overall survival.P<0.05*; P<0.01**; P<0.001***

Supplemental Table 7 PFS-relevant variable data for external patients

| **Variable** | **All patients in the external**  **cohort (N = 50)** | **Non- Progressive**  **（N=39）** | **Progressive（N=11）** | **P Value** |
| --- | --- | --- | --- | --- |
| **Lymphatic metastasis**  No  Yes | 39（78.00%）  11（22.00%） | 35（89.74%）  4（10.26%） | 4（36.36%）  7（63.64%） | 0.001** |
| **Radiotherapy**  No  Yes | 48（96.00%）  2（4.00%） | 39（100.00%）  0（0%） | 9（81.82%）  2（18.18%） | 0.045* |
| **Antibacterials**  No  Yes | 1（2.00%）  49（98.00%） | 1（2.56%）  38（97.44%） | 0（0%）  11（100%） | 1 |
| **NLR**  <3.470  ≥3.470 | 41（82.00%）  9（18.00%） | 34（87.18%）  5（12.82%） | 7（63.64%）  4（36.36%） | 0.177 |
| **SIRI**  <1.66  ≥1.66 | 40（80.00%）  10（20.00%） | 36（92.31%）  3（7.69%） | 4（36.36%）  7（63.64%） | 0.000*** |

**Abbreviations:** NLR, neutrophil-to-lymphocyte ratio; SIRI, systemic inflammation response index; PFS, progression-free survival.P<0.05*; P<0.01**; P<0.001***
